# The spatial distribution of known predictors of autism spectrum disorders impacts geographic variability in prevalence in central North Carolina

**DOI:** 10.1186/1476-069X-11-80

**Published:** 2012-10-31

**Authors:** Kate Hoffman, Amy E Kalkbrenner, Veronica M Vieira, Julie L Daniels

**Affiliations:** 1 University of North Carolina, Gillings School of Global Public Health, CB #7435, Chapel Hill, NC, 27599, USA; 2 University of Wisconsin at Milwaukee, Zilber School of Public Health, 3230 E. Kenwood Blvd, Milwaukee, WI, 53211, USA; 3 Boston University School of Public Health, 715 Albany St, Boston, MA, 02118, USA; 4 School of Ecology, University of California, Irvine, CA, 92617, USA

**Keywords:** Autism spectrum disorders (ASD), Intellectual disability (ID), Spatial analysis, Disease mapping, Generalized additive models (GAMs), Geographic information systems (GIS)

## Abstract

**Background:**

The causes of autism spectrum disorders (ASD) remain largely unknown and widely debated; however, evidence increasingly points to the importance of environmental exposures. A growing number of studies use geographic variability in ASD prevalence or exposure patterns to investigate the association between environmental factors and ASD. However, differences in the geographic distribution of established risk and predictive factors for ASD, such as maternal education or age, can interfere with investigations of ASD etiology. We evaluated geographic variability in the prevalence of ASD in central North Carolina and the impact of spatial confounding by known risk and predictive factors.

**Methods:**

Children meeting a standardized case definition for ASD at 8 years of age were identified through records-based surveillance for 8 counties biennially from 2002 to 2008 (n=532). Vital records were used to identify the underlying cohort (15% random sample of children born in the same years as children with an ASD, n=11,034), and to obtain birth addresses. We used generalized additive models (GAMs) to estimate the prevalence of ASD across the region by smoothing latitude and longitude. GAMs, unlike methods used in previous spatial analyses of ASD, allow for extensive adjustment of individual-level risk factors (e.g. maternal age and education) when evaluating spatial variability of disease prevalence.

**Results:**

Unadjusted maps revealed geographic variation in surveillance-recognized ASD. Children born in certain regions of the study area were up to 1.27 times as likely to be recognized as having ASD compared to children born in the study area as a whole (prevalence ratio (PR) range across the study area 0.57-1.27; global *P*=0.003). However, geographic gradients of ASD prevalence were attenuated after adjusting for spatial confounders (adjusted PR range 0.72-1.12 across the study area; global *P*=0.052).

**Conclusions:**

In these data, spatial variation of ASD in central NC can be explained largely by factors impacting diagnosis, such as maternal education, emphasizing the importance of adjusting for differences in the geographic distribution of known individual-level predictors in spatial analyses of ASD. These results underscore the critical importance of accounting for such factors in studies of environmental exposures that vary across regions.

## Background

Autism spectrum disorders (ASD) are complex neurodevelopmental disorders characterized by impaired social interaction and communication, and restrictive and repetitive behavior [1]. Estimates in 2008 indicate that approximately 1 in 88 children have ASD and that the prevalence of documented ASD is on the rise [2]. The causes for ASD remain largely unknown and widely debated [3,4]. Environmental exposures are hypothesized to contribute to ASD etiology [5]; however, identifying exposures of concern has been complicated by the relative rarity of ASD, the extensive number of candidate exposures, and the lack of exposure measurements during early life, the developmentally relevant time period for ASD. Thus, studies have explored the geographic distribution of ASD as a means for generating hypotheses about spatially distributed environmental exposures. Additionally, studies have used geographically-based exposure assignment to evaluate the impact of specific exposures such as air pollutants, pesticides, and hazardous wastes on ASD risk [6-9].

One problem with evaluating the geographically non-random prevalence of ASD for etiologic purposes lies in difficulties disentangling the geographic distribution of other factors associated with diagnosis [10,11]. For example, higher maternal education is associated with increased ASD diagnosis in the United States [12,13] but not in some European countries [14]. These results suggest that maternal education is a factor in promoting recognition of ASD, but not necessarily the occurrence of ASD. Identifying patterns related to ASD diagnosis may be helpful for public health services allocation; however, to generate hypotheses about etiology, we must distinguish diagnostic patterns from patterns of ASD occurrence. For example, prioritizing investigations of a geographically-based environmental cause may be unwarranted if the observed spatial pattern is driven by maternal education (i.e. spatial confounding).

Two previous studies investigated geographic variability of ASD as a means of identifying environmental exposures related to ASD prevalence [15,16]. Both reported that children born in certain regions of California were more likely to have a recognized ASD than children born in other parts of the state [15,16]. The authors attributed their findings to regional differences in the underlying population (i.e. demographic and socioeconomic factors) or geographic variability of environmental exposures; but were unable to disentangle factors promoting diagnosis from environmental exposures because they did not adjust for potentially important individual-level spatial confounding [15,16].

In order to determine the potential for environmentally distributed exposure to be associated with ASD in central North Carolina (such as contaminants in air or water), we first explored whether spatial differences in ASD prevalence existed and then whether differences remained after accounting for spatially distributed covariates associated with ASD risk and diagnosis. We used a method of spatial epidemiology that applies generalized additive models (GAMs) to assess the spatial variation of disease in a region while simultaneously adjusting for other geographically distributed individual-level factors [17,18] such as maternal education, age, and smoking. Combining GAMs and geographic information systems allowed us to predict a continuous surface of ASD prevalence across our study area. Our research serves not only to expand the consideration of spatial patterns of ASD to geographic regions other than California but also to improve the utility of such studies by directly examining how adjustment for known risk and predictive factors influences geographic patterns. Patterns remaining after accounting for factors that influence ASD recognition may better reveal the distribution of novel geographically distributed etiologic factors impacting ASD prevalence.

## Methods

### Study population

The Autism and Developmental Disabilities Monitoring (ADDM) Network is an active, population-based surveillance program that monitors the prevalence of developmental disabilities among children aged 8 years, an age by which most children with ASD have been evaluated [19], in selected geographic regions across the United States [20]. The ADDM Network conducts standardized review of medical and educational records and trained clinicians determine whether standardized case definitions for ASD and intellectual disability (ID) are met [20]. Our analyses utilized data from the North Carolina ADDM site (NC-ADDM), which began biennial surveillance in 2002. Analyses were restricted to children born in the 8 counties that were under surveillance during all study years (2002–2008).

**Figure 1:**
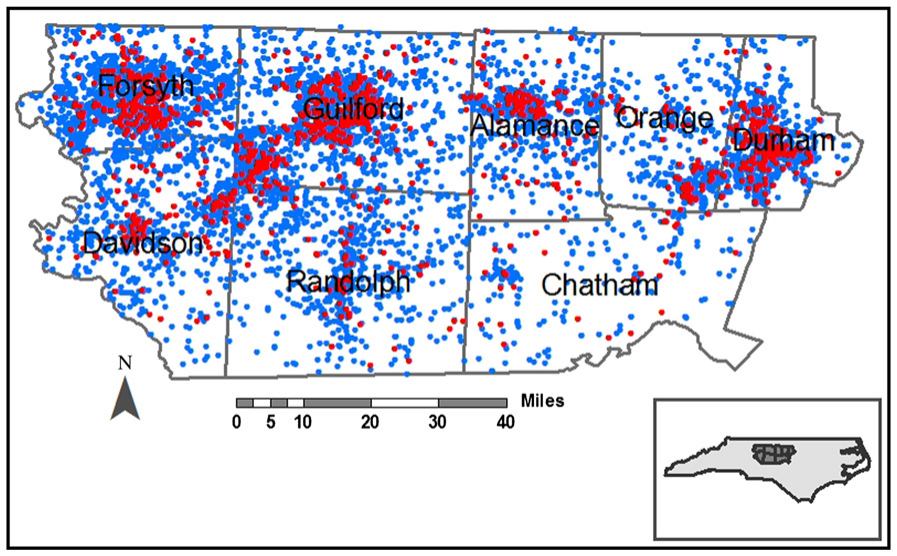
**Eight county central North Carolina study area.** The residential addresses at birth for the birth cohort (blue points) and children with ASD or ID (red points) born in 1994, 1996, 1998 and 2000 are displayed with altered locations to preserve confidentiality.

To represent the underlying population, we randomly selected a 15% sample of birth records for children born in the same study counties and years as children included in ADDM (biennial 1994–2000; n=11,908, representing a region averaging 20,000 births per year). Figure 1 provides orientation to the population distribution in NC, where red dots indicate children with developmental disabilities (ASD or ID) and blue dots indicate children randomly sampled from the entire central NC area. We excluded children who were adopted because information on birth address was missing and those who died in infancy because they were not part of the risk set for development disabilities (n=93; <1%). NC-ADDM and our current analyses were reviewed and approved by the Institutional Review Board at the University of North Carolina-Chapel Hill.

### ASD and ID classification

To fully explore the impact of spatial confounding on the geographic distribution of developmental disability, we considered 4 different disability groups: all children with an ASD, 2 subgroups within all ASD classified by the co-occurrence of ID (ASD+ID and ASD-ID), and the independent group of all children with ID without regard to ASD status. Examining these independent and cross-classified groups provided a more nuanced disentanglement of factors which may act differently to promote ASD, versus ID, recognition, as follows. Diagnosis of ASD requires comprehensive evaluation of a constellation of behaviors that can be more involved than a singular evaluation assessment of Intelligence Quotient (IQ) to determine ID. Yet, ASD often occurs with ID, frequently presenting more severe disability that is recognized at an earlier age. In addition, Durkin et al. (2010) reported a differential socioeconomic (SES) gradient in ASD prevalence by the presence or absence of ID; SES acts as a stronger risk factor for ASD-ID compared to ASD+ID [3]. Because we expect SES to vary spatially and be a spatial confounder, analyzing ASD without respect to ID may mask the true spatial patterning of disease.

Children met the standardized case definition for ASD if clinician reviewers determined their developmental evaluation records indicated behaviors consistent with ASD, based on the Diagnostic and Statistical Manual IV^TM^ criteria for Autistic Disorder, Asperger Disorder, or Pervasive Developmental Disorder Not-Otherwise-Specified [21]. Children met the standardized definition for ID if clinician review of developmental evaluations determined they had an IQ ≤ 70 on the most recently administered psychometrics test such as the Battelle–cognitive domain [22], Differential Ability Scales [23], Stanford-Binet–4th ed. [24], Wechsler Preschool and Primary Scale of Intelligence [25], and the Wechsler Intelligence Scale for Children-III [26], or, in the absence of test scores, a written statement in the records indicated the presence of a severe or profound intellectual disability [20]. Our analyses included 561 children with ASD, who were further classified into two subgroups: children with ASD without ID (ASD-ID, n=330), and children with ASD and ID (ASD+ID, n=231). As a comparison to the ASD analyses, we conducted additional analyses investigating the spatial variability in the prevalence of ID (n=1,028) regardless of ASD status.

### Residential location and covariates

Surveillance data were linked to birth records to obtain the residential address and covariate information at the time of birth of children with ASD and ID. Birth data were chosen to reflect the most etiologically relevant time period for brain development [27].

We combined several methods to geocode (i.e. assign latitude and longitude coordinates) the residential birth addresses of study children to improve geocoding accuracy and reduce positional errors [28]. First, we ran all addresses through the ZP4 software which cleans and updates addresses using U.S. Postal Service databases (e.g. converting rural routes to E-911 street names) (version expiring March 2011, Semphoria; Memphis Tennessee). We then cleaned all addresses individually and geocoded them using ArcGIS (version 9.3, Redlands, California) and U.S. Census Tigerline files [29]. For unmatched addresses, we used Google Maps to locate the residence where possible [30]. Using these methods, we successfully geocoded 12,299 (93.41%) of the 13,167 residential addresses. Of the addresses we were unable to geocode, 379 (2.88%) were post office boxes and 489 (3.71%) were addresses that were either incomplete or that we were not able to geocode to a specific location. Geocoding success was similar for the birth cohort and children with ASD and ID.

### Spatial analysis

We estimated the log odds of ASD and ID using GAMs, an extension of linear regression models that can analyze binary outcome data and accommodate both parametric and non-parametric model components [17]. For non-parametric model terms, GAMs replace the traditional exposure term in an ordinary logistic regression with a smooth term (i.e. a term of best fit after adjusting for other covariates). In these analyses we applied a bivariate smooth to latitude and longitude coordinates and included all other covariates as parametric terms [17,18].

We used a locally weighted regression smoother (loess) which adapts to changes in the data density that are likely to occur in analyses of residential locations due to variability in population density. To predict prevalence the loess smoother utilizes information from nearby data points (weighting information based on its distance from the prediction point). The region or neighborhood from which data are drawn to predict prevalence is based on the percentage of data points in the neighborhood and is referred to as the span size. Choice of span size is a trade-off between bias and variability. A larger span size (more data included) results in a flatter surface with low variability but increased bias, while a small span size results in high variability and comparatively low bias. We determined the optimal amount of smoothing (optimal span size), by minimizing Akaike’s Information Criterion (AIC) [17,18]. A large optimal span size indicates less spatial variability in prevalence compared to a small optimal span.

We created a grid covering the study area that extended across all latitude and longitude coordinates of all addresses. We excluded grid points in regions of the study area where no children in our sample were born. The resulting grid comprised approximately 4,300 points. We predicted the log odds for each of the 4 developmental outcome groups (e.g. ASD) at each point on the grid and calculated an odds ratio using the entire study area as the referent group; the odds at each point was divided by the odds from a reduced model which omitted the latitude and longitude smooth term [18]. We did not remove children with developmental outcomes from the random selection of births drawn to represent the underlying distribution of births and, consequently, some cases were included in the denominator. As a result, the odds ratios from these models are mathematically equivalent to prevalence ratios. We mapped prevalence ratios using a continuous color scale (blue to red) and a constant scale range for all maps with the same outcome (i.e. ASD; ASD+ID; ASD-ID; and ID).

To provide information in the interpretation of spatial patterns that could be driven by sparse data, we preformed a 2-step statistical screening procedure, as follows. First, we tested the null hypothesis that developmental disability does not depend on geographic location, generally (e.g. the predicted map surface is a flat horizontal plane). Residential locations of individuals were permuted 999 times while preserving their case status and covariates [as described in [20]. In each permutation, the GAM with the optimal span size from the original dataset was run and the global deviance statistic computed. We used a conservative p<0.025, which accounts for inflated type 1 error rates associated with using the optimal span size for the original dataset in permutations, to determine if location was a statistically significant predictor of disability [details in [31]. If the global statistic indicated that location was a statistically significant predictor of disability generally, we next evaluated location-specific (point-wise) departures from the null hypothesis using the same set of permutations [18,31]. Regions with significantly increased or decreased surveillance-recognized ASD or ID prevalence were defined as points ranking in the upper or lower 2.5% of the distribution of permuted prevalence ratios at each point, respectively [18].

Statistical analyses were performed in the R Package 2.12.02 (Vienna, Austria) using the gam library and a local scoring algorithm GAM estimation procedure and publically available statistical code [32,33]. All maps were created using ArcGIS 9.3 (version 9.3, Redlands, California).

### Confounding

We adjusted models for several previously established ASD predictive factors including year of birth; plurality; maternal age, race/ethnicity, and level of education; and report of tobacco use during pregnancy (categorizations in Table 1, [3,11,12,34-36]). Covariate data were nearly complete for these variables; however 35 children (<1%) had missing data and were excluded from all analyses. Additionally, we investigated confounding by other factors potentially related to ASD risk or recognition including method of delivery (vaginal delivery vs. cesarean section), marital status (married vs. unmarried), birth weight (<2500 g; 2501–3000 g; 3001–4000 g; >4000 g), and adequacy of prenatal care [assessed using the Adequacy of Prenatal Care Utilization Index; categorized as less than adequate (inadequate or intermediate prenatal care); adequate; adequate-plus [37]]. We used 3 approaches to fully assess the confounding influence of these factors: 1) We assessed the change in patterns of spatial variability, using a side by side visual inspection of maps before and after adjustment, comparing the areas of reduced and elevated prevalence ratios and the color intensities (indicating the magnitude of prevalence ratios). To assure equivalency, the number of observations and span size were held constant [18]. 2) We also investigated changes in the model-selected optimal span size of analyses with and without adjustment. A smaller optimal span size, using less of the data, is selected when the data supports more peaks and valleys in prevalence. It follows that a change in the optimal span size after the inclusion of a covariate can indicate spatial confounding. 3) Finally, to investigate the spatial variability of the predictive factors themselves (spatial associations between the factor and disability, necessary to cause confounding), we compared maps of each covariate to maps of the developmental disability.

**Table 1:** Selected Characteristics of the Birth Cohort and Children with ASD and ID in Eight North Carolina Counties in 2002, 2004, 2006 and 2008

**Variable**	**Birth Cohort n (%)**	**All ASD n (%)**	**ASD-ID n (%)**	**ASD+ID n (%)**	**All ID n (%)**
**Total**	11809 (100.00)	561 (100.00)	330 (100.00)	231 (100.00)	1028 (100.00)
**Sex**					
**Male**	6073 (51.43)	464 (82.71)	279 (84.55)	185 (80.09)	665 (64.69)
**Female**	5736 (48.57)	97 (17.29)	51 (15.45)	46 (19.91)	363 (35.31)
**Year of Birth**					
**1994**	2727 (23.09)	87 (15.51)	49 (14.85)	38 (16.45)	224 (21.79)
**1996**	2825 (23.92)	119 (21.21)	66 (20.00)	53 (22.94)	266 (25.88)
**1998**	2958 (25.05)	151 (26.92)	86 (26.06)	65 (28.14)	254 (24.71)
**2000**	3299 (27.94)	204 (36.36)	129 (39.09)	75 (32.47)	284 (27.63)
**Maternal Age at Birth**					
**Under 25**	4984 (42.21)	184 (32.80)	89 (26.97)	95 (41.13)	460 (44.75)
**25-35**	5435 (46.02)	281 (50.09)	184 (55.76)	97 (41.99)	465 (45.23)
**Over 35**	1388 (11.75)	96 (17.11)	57 (17.27)	39 (16.88)	103 (10.02)
**Missing**	2 (0.02)	0 (0.00)	0 (0.00)	0 (0.00)	0 (0.00)
**Maternal Race**					
**White**	8148 (69.00)	368 (65.6)	238 (72.12)	130 (56.28)	519 (50.49)
**Other**	3661 (31.00)	193 (34.4)	92 (27.88)	101 (43.72)	509 (49.51)
**Maternal Educational Attainment**					
**Less than High School**	2553 (21.62)	76 (13.55)	29 (8.79)	47 (20.35)	377 (36.67)
**High School**	3424 (29.00)	149 (26.56)	66 (20.00)	83 (35.93)	372 (36.19)
**Some College**	2472 (20.93)	126 (22.46)	77 (23.33)	49 (21.21)	144 (14.01)
**College or More**	3341 (28.29)	208 (37.08)	157 (47.58)	51 (22.08)	134 (13.04)
**Missing**	19 (0.16)	2 (0.36)	1 (0.30)	1 (0.43)	1 (0.10)
**Maternal Tobacco Use During Pregnancy**					
**Yes**	1647 (13.95)	67 (11.94)	38 (11.52)	29 (12.55)	208 (20.23)
**No**	10150 (85.95)	493 (87.88)	292 (88.48)	201 (87.01)	819 (79.67)
**Missing**	12 (0.10)	1 (0.18)	0 (0.00)	1 (0.43)	1 (0.10)
**Plurality**					
**Yes**	353 (2.99)	23 (4.10)	14 (4.24)	9 (3.90)	54 (5.25)
**No**	11456 (97.01)	538 (95.90)	316 (95.76)	222 (96.10)	974 (94.75)

### Robustness of analyses

Our final dataset contained some siblings. In addition to being genetically more similar to each other, siblings typically share the same residence. Including siblings living at the same address in analyses could induce spatial clustering as a result of familial (i.e. genetic) similarities rather than geographically-linked factors. To assess the robustness of our results to including a small number of sibling groups, we conducted secondary analyses including only one randomly selected child per family. Families were defined as children for whom the mother had the same first and maiden name and date of birth (obtained from birth certificates). Because information for fathers was missing and incomplete on many birth records, we did not attempt to identify paternal siblings.

## Results

Selected characteristics for the birth cohort and children with ASD or ID are displayed in Table 1. Children with ASD were more likely to be male, have older mothers, and mothers with higher educational attainment. As has been reported previously [2,38], the age 8 prevalence of ASD in the region increased from 2002–2008, however the prevalence of ID remained relatively stable (Table 1). Residential locations at birth for our study population are displayed in Figure 1 with slight alteration to preserve confidentiality.

**Figure 2:**
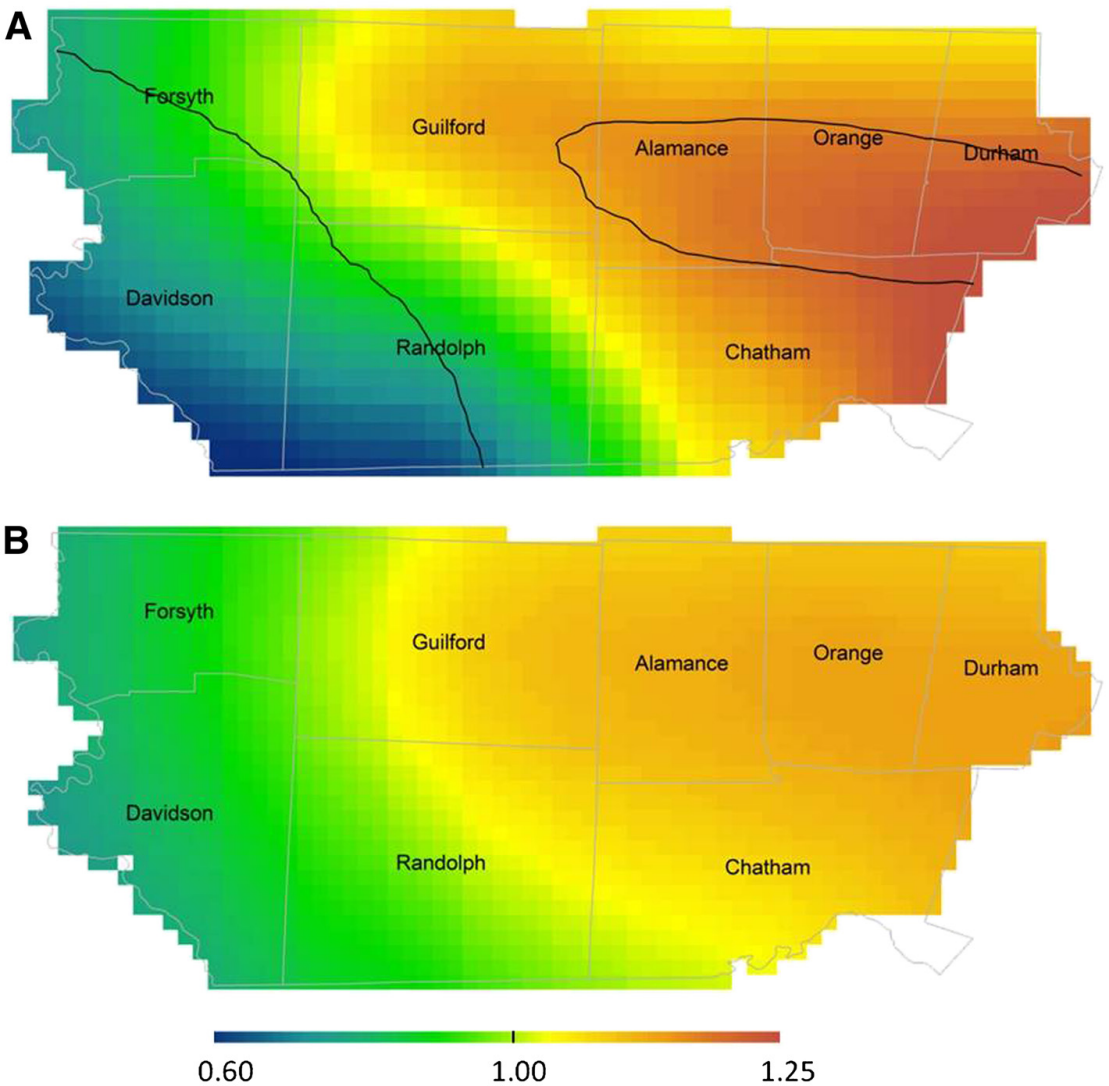
**Geographic distribution of ASD prevalence relative to the birth cohort n=11,034 and ASD n=532: Unadjusted (A) and fully adjusted models (B) are presented using the optimal span size of each (0.75 and 0.95 respectively).** The unadjusted model is significantly different than flat (global *P*=0.003). Areas of significantly increased and decreased prevalence are indicated by black contour bands. Adjusted model is not significantly different than flat (global *P*=0.052). Adjustment factors were year of birth; plurality; maternal age, race, and level of education; and report of tobacco use during pregnancy.

We found geographic variability in ASD prevalence across the study area in unadjusted analyses, as indicated by the global statistical test (Figure 2a; optimal span size=0.75; global *P=*0.003; Table 2). The Point-wise statistical tests identified areas of increased ASD prevalence in portions of Alamance, Durham, and Orange Counties, and children living in these areas were 1.10 to 1.27 times as likely to have a surveillance-recognized ASD at age 8 years compared to children born in the study area as a whole. Conversely, children born in the western part of the region were 0.57 to 0.90 times as likely to have a surveillance-recognized ASD. Geographic variability in ASD prevalence was attenuated after adjusting for confounding by year of birth; plurality; maternal age, race, and level of education; and report of tobacco use during pregnancy, indicated by the following results. In the adjusted model, the optimal span size (determined by minimizing the model AIC) increased, indicating less variability, and the global p-value was not consistent with departures from a flat pattern of ASD prevalence (Figure 2b; optimal span=0.95; global *P*=0.052; Table 2). The range of prevalence ratios across the study area was diminished: the adjusted model yielded PRs ranging from 0.72 to 1.12, in contrast to the unadjusted model, where PRs ranged from 0.57 to 1.27.

**Table 2:** Summary of Spatial Analyses

**Variable**	**All ASD (birth cohort n=11,034 and ASD n=532)**		**ASD-ID (birth cohort n=11,034 and ASD-ID n=318)**		**ASD+ID (birth cohort n=11,034 and ASD+ID n=214)**		**All ID (birth cohort n=11,034 and ID n=916)**	
	**Unadjusted**	**Adjusted**	**Unadjusted**	**Adjusted**	**Unadjusted**	**Adjusted**	**Unadjusted**	**Adjusted**
PR Range	0.57-1.27	0.72-1.12	0.63-1.66	0.73-1.22	0.49-1.40	0.56-1.20	0.12-2.23	0.44-1.75
Span Size	0.75	0.95	0.70	0.95	0.95	0.95	0.10	0.30
Global p-value	0.003	0.052	0.027	0.294	0.041	0.196	<0.001	0.065
Figure	2a	2b	--	AF 2b	--	AF 2c	3a	3b

AF – Additional File.

Additionally adjusting for method of delivery, marital status, birthweight, and adequacy of prenatal care did not change the appearance of maps for any of the 4 developmental disability groups considered, or the range of prevalence ratios observed across the study area; these variables were dropped from adjusted analyses. Spatial confounding was driven primarily by the higher educational attainment in Alamance, Chatham, Durham, and Orange Counties, and to a lesser extent, to greater maternal age observed in the same counties; maps with adjustment for maternal age and education only had the same optimal span size (0.95), a similar range of prevalence ratios, and were visually very similar to the fully adjusted maps. When we examined the patterns of maternal educational attainment and age across the study area, both were similar to the unadjusted ASD pattern. For example, mothers living in the areas where the unadjusted ASD prevalence was highest were approximately 1.75 times as likely to have completed college than women in the study area as a whole (Additional file 1: Figure S1).

Among the 532 children with ASD, 214 (40.22%) also had an ID. The spatial patterns of both subgroups (ASD+ID and ASD-ID) were similar to each other and to the map of the combined group (all ASD); the locations of increased prevalence and the intensity of the increases were similar across all maps (Table 2; Additional file 1: Figure S2). Adjusting for spatial confounding resulted in flatter maps of ASD+ID and ASD-ID; however, greater attenuation was observed in the analysis of ASD-ID in which the optimal span size increased from 0.70 to 0.95 after adjustment (indicating the surface was less variable/ more flat after adjustment; Table 2; unadjusted figures not shown). Additionally, the range of PRs was more substantially attenuated by adjustment in the analysis of ASD-ID. In our sample, ASD-ID was 1.33 times as high in children who had mothers with college or more education (referent group=children of mothers with some post secondary education; *P*=0.059); but these same mothers were less likely than mothers with some postsecondary education to have a child with ASD + ID (aPR = 0.67, *P* = 0.060).

**Figure 3:**
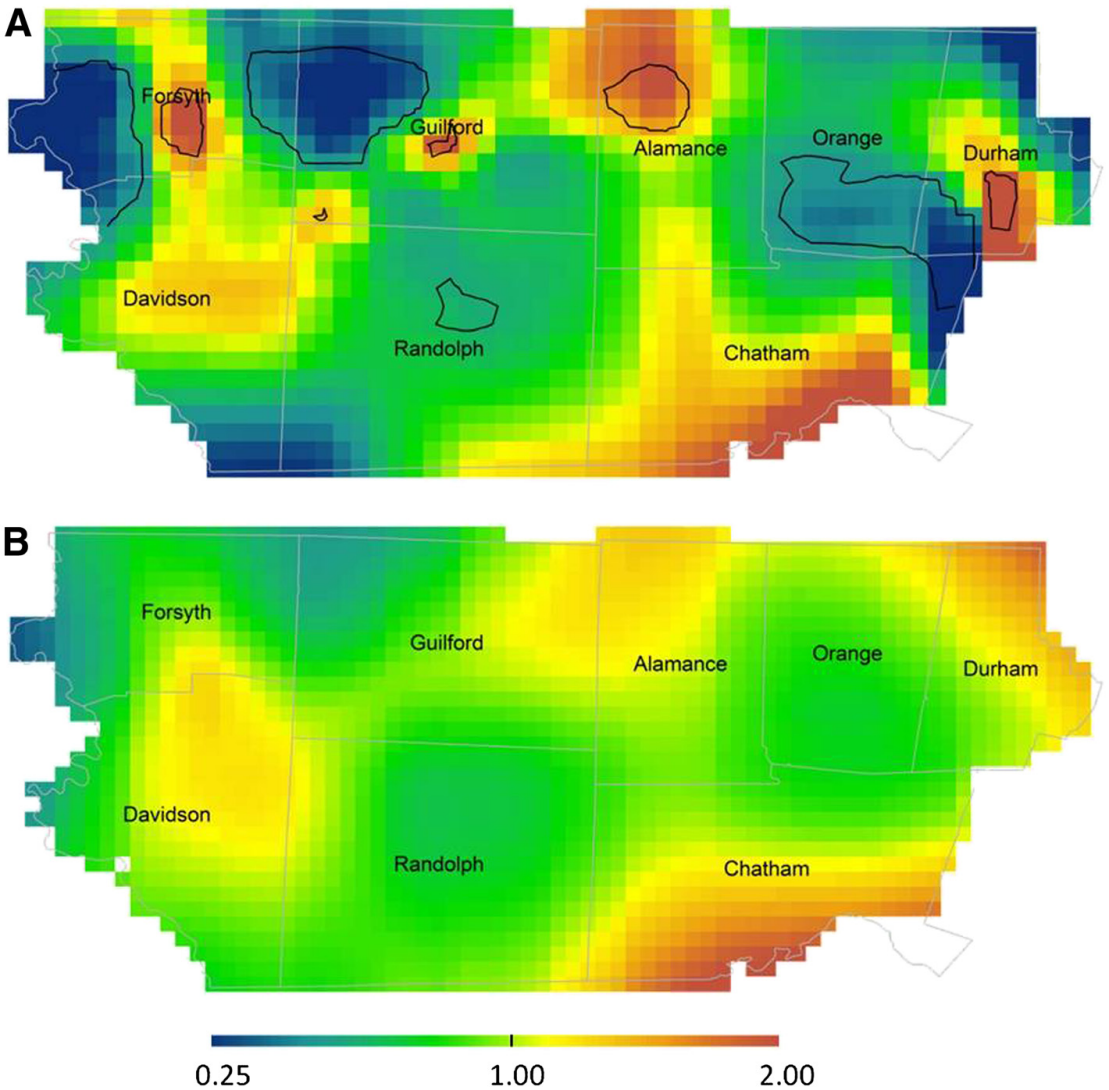
**Geographic distribution of ID prevalence relative to the birth cohort n=11,034 and ID n=916: Unadjusted (A) and fully adjusted models (B) are presented using the optimal span size of each (0.10 and 0.30 respectively).** The unadjusted model is significantly different than flat (global *P*<0.001). Areas of significantly increased and decreased risk are indicated by black contour bands. Adjusted model is not significantly different than flat (global *P*=0.065). Adjustment factors were year of birth; plurality; maternal age, race, and level of education; and report of tobacco use during pregnancy.

When ID was analyzed as a separate disability, unadjusted analyses revealed significant spatial variability in prevalence across the study area; several areas of increased and decreased ID prevalence were observed (Figure 3a; optimal span = 0.10; global *P* < 0.001). Geographic gradients in prevalence ratios were somewhat attenuated in the adjusted analyses, however, patterns of residual variability in the prevalence of ID remained after accounting for known predictors (Figure 3b; optimal span = 0.30; global *P* = 0.065). Additionally, although adjusting for spatial confounding resulted in a larger span size, the optimal span size of the adjusted analyses remained small (0.30), indicating spatial variability; however the adjusted analyses did not reach global statistical significance at the alpha = 0.025 level.

Only 269 sibling pairs were in our analyses and their impact on analyses of both ASD and ID was negligible. When we randomly selected one child from each family for analyses, the pattern of spatial variability was quite similar to analyses including all children (results not shown).

## Discussion

Although we observed spatial variability in ASD prevalence in unadjusted analyses, the pattern of ASD appeared to be largely explained by factors influencing diagnosis -- maternal age and education -- which differed across the study area. The larger optimal span size and global p-value in the adjusted analyses, as well as decreased variability in prevalence ratios across the study area, all indicated less geographic variability after adjustment for known risk and predictive factors for ASD. Our results corroborate previous reports [15,16] demonstrating spatial variability in ASD prevalence before adjusting for spatial confounding. In secondary analyses Van Meter and colleagues reported the impact of living in neighborhoods with high ASD prevalence was diminished in analyses adjusting for parental education, a finding consistent with our results [16].

Our results highlight the importance of adjusting for the geographic distribution of known individual-level predictors in spatial analyses. Here, adjustment for socioeconomic factors associated with diagnosis greatly attenuated geographic variability in ASD prevalence. This has implications for studies of ASD etiology that assign environmental exposures based on geography, such as air pollutants and agricultural pesticides, for which we caution the causal interpretation of geographic patterns that are not controlled for individual-level factors. Using GAMs allowed us to carefully account for known risk and diagnostic factors, a major strength of these analyses [18]. In addition, we considered both the statistical and qualitative aspects of the observed geographic patterns, tempering interpretation of areas of sparse data by considering tests of global deviance and changes in the optimal span size along-side visual inspection of the patterns in our interpretation of results.

NC-ADDM Network provided a large population based sample with information on co-occurring conditions, allowing us to consider differences in spatial confounding by the severity and type of disability. Patterns for separate disability groups, ASD+ID and ASD-ID, were visually similar to those for all ASD; however, ASD-ID appeared to be more impacted by spatial confounding as indicated by a greater attenuation in prevalence ratios and a increase of the optimal span size in the adjusted analysis. The associations between ASD (+/−ID) and maternal education and age that we observed in these analyses are consistent with previously reported associations in non-spatial analyses [3,4] which indicate that ASD-ID is more strongly associated with higher SES than ASD+ID.

Although the global significance test did not indicate variability, our results suggest that ID prevalence varied geographically across the study area; even after adjusting for known spatial confounders the optimal span size remained small and results were visually suggestive of geographic differences in ID prevalence. There are several possible explanations for the observed variability in ID, including residual confounding by unmeasured variables and differences in the distribution of environmental factors across the study area.

Linking data from the NC-ADDM Network and vital records also strengthened our analyses. Vital records provided individual-level data on a number of covariates, allowing us to account for spatial confounding by these factors, and also provided residential location at the time of birth, which may be more relevant to ASD etiology than the address at diagnosis. Although the majority of children with ASD at age 8 in ADDM were also born in the study area, local changes in address were common (68.14% of children with ASD had a change in residential address between birth and age 8). If the spatial patterns we observed are due to difference in diagnosis, spatial variability may be more apparent using a later address corresponding to the time of diagnosis.

A final strength of our study was our ability to evaluate the influence of potential sibling clusters in analyses. While it is often suggested that including siblings in analyses will induce clustering, we did not observe this pattern in our data. One possible explanation is that the number of siblings in our analyses was relatively small (e.g. 269 sibling pairs in the ASD analysis of 11,566 children), due in part to the biennial study design and the selection of only part of the source population (15%).

There are also several important limitations of our methods. We chose the optimal tradeoff between bias and variance of the smooth (the optimal span size) by minimizing the AIC. Selecting the optimal span size for a dataset, however, may obscure important small scale variability in maps; it is possible that our analyses were conducted on a scale too large to identify small scale environmental exposures relevant in the etiology of ASD. Examining different span sizes may reveal important features of the data. Similarly, we assessed spatial confounding by visually comparing maps of prevalence before and after adjustment holding the span size and included observations constant and by investigating changes in the optimal span size before and after adjustment [18]; however, more objective methods of assessing confounding are needed and are an important topic for future research. We also used p-values as a screening tool, to evaluate the global spatial variation as well as areas of increased or decreased prevalence. Our use of p-values here was to evaluate whether or not spatial variability existed (i.e. whether the prevalence of ASD was constant across the geographic area). Nonetheless, many epidemiologists prefer the use of confidence intervals, which provide information on the precision of the observed association [39]. While it is possible to calculate confidence intervals in these analyses, it is visually difficult to display three surfaces simultaneously on maps. Additionally, we conducted permutation tests using the span size of the observed data to test the null hypothesis that the map is flat. Permuted datasets under this null hypothesis may have had a larger optimal span size, particularly for the ID analyses, which had a relatively small span size for the observed data (optimal span = 0.30). Consequently, using the span size of the observed data could result in a p-value that is too small [18].

## Conclusions

Our results demonstrate the importance of adjusting for predictive and diagnostic factors that may spatially confound the search for novel environmentally-distributed risk factors for ASD. These adjustment methods can help the search for causes of developmental disabilities proceed more efficiently by aiding the interpretation of geographic patterns. Cumulatively, our results suggest that known predictive factors for ASD account for a large portion of the observed geographic variability in prevalence in central North Carolina. Although we did not identify spatial variability in ASD in NC after controlling for known predictive and risk factors, follow up of these results in other regions is may provide clues for ASD etiology.

## Abbreviations

AF: Additional file; APR: Adjusted prevalence ratio; ADDM Network: Autism and developmental disabilities monitoring network; AIC: Akaike’s information criterion; ASD: Autism spectrum disorders; ASD+ID: Autism spectrum disorders with intellectual disability; ASD-ID: Autism spectrum disorders without intellectual disability; ID: Intellectual disability; GAM: Generalized additive model; GIS: Geographic information systems; PR: Prevalence ratio; SES: Socioeconomic status.

## Competing interests

All authors declare that they have are no actual or potential competing interests.

## Authors’ contributions

KH assisted in conceiving the study, conducted analyses, and drafted the manuscript. AK assisted conceiving the study and the analysis plan, and assisted in manuscript editing. VV collaborated on statistical issues, and assisted in the analysis plan and manuscript editing. JD, the principle investigator of the North Carolina Autism and Developmental Disabilities Monitoring Network, assisted in the analysis plan and manuscript editing. All authors read and approved the final manuscript.

## Supplementary Material

Additional file 1 **Figure S1.** Mother's educational attainment at the time of birth n=11,034. Map reflects the optimal span size of the ASD analyses (span=0.95; global *P*<0.001); the optimal span size of the education analysis was 0.05 (global *P*<0.001). Larger prevalence ratios indicate a higher prevalence of mothers with college or more education at the child’s birth. Areas of significantly increased and decreased risk are indicated by black contour bands. **Figure S2.** Adjusted maps for (A) ASD prevalence (birth cohort n=11,034 and ASD n=532), (B) ASD-ID (birth cohort n=11,034 and ASD-ID n=318), and (C) ASD+ID (birth cohort n=11,034 and ASD+ID n=214). Maps are not significantly different than flat (global *P*=0.052, global *P*=0.294 and global *P*=0.196, respectively). Adjustment factors were year of birth; plurality; maternal age, race, and level of education; and report of tobacco use during pregnancy. Click here for file
