# Supplementary material for: The spatial distribution of known predictors of autism spectrum disorders impacts geographic variability in prevalence in central North Carolina
Source: Environ Health. 2012 Oct 31;11:80. doi: 10.1186/1476-069X-11-80 (PMC3499188; doi:10.1186/1476-069X-11-80)

**Title:** Autism spectrum disorder in central North Carolina: A spatial analysis using generalized additive models

**Figure 1……………………………………………………………………………………..Page 2**

Mother’s educational attainment at the time of birth n=11,034. Map reflects the optimal span size of the ASD analyses (span=0.95; global *P*<0.001); the optimal span size of the education analysis was 0.05 (global *P*<0.001). Larger prevalence ratios indicate a higher prevalence of mothers with college or more education at the child’s birth. Areas of significantly increased and decreased risk are indicated by black contour bands.

**Figure 2……………………………………………………………………………………..Page 3**

Adjusted maps for (A) ASD prevalence (birth cohort n=11,034 and ASD n=532), (B) ASD-ID (birth cohort n=11,034 and ASD-ID n=318), and (C) ASD+ID (birth cohort n=11,034 and ASD+ID n=214). Maps are not significantly different than flat (global *P*=0.052, global *P*=0.294 and global *P*=0.196, respectively). Adjustment factors were year of birth; plurality; maternal age, race, and level of education; and report of tobacco use during pregnancy.

Figure 1:


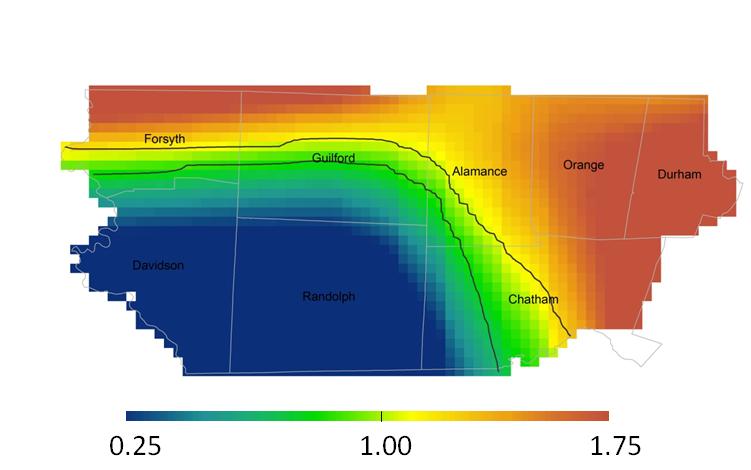


Figure 2:


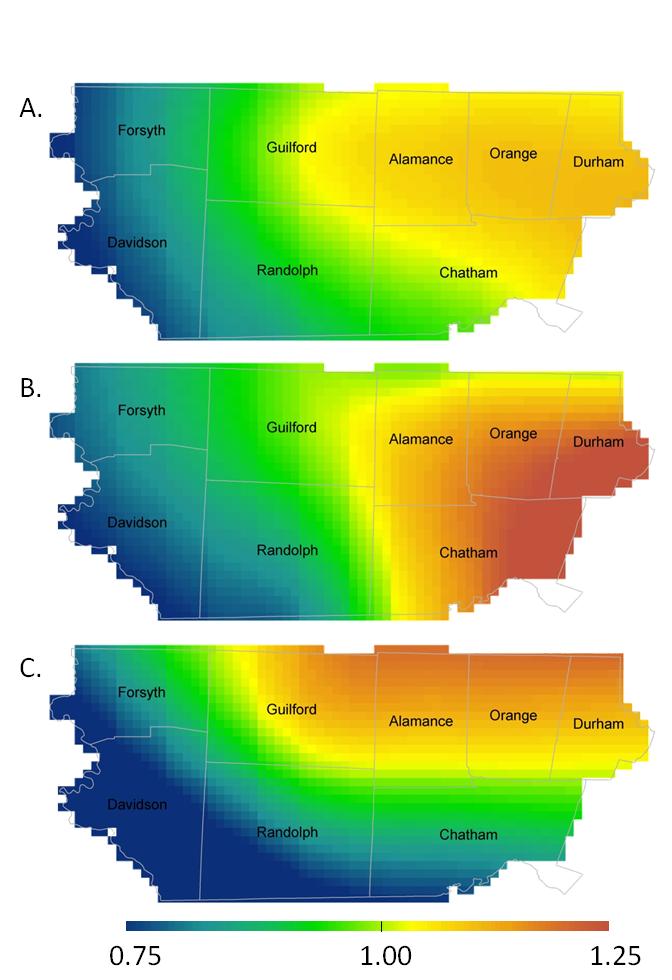

Supplement: Additional file 1 — Figure S1. Mother's educational attainment at the time of birth n=11,034. Map reflects the optimal span size of the ASD analyses (span=0.95; global P<0.001); the optimal span size of the education analysis was 0.05 (global P<0.001). Larger prevalence ratios indicate a higher prevalence of mothers with college or more education at the child’s birth. Areas of significantly increased and decreased risk are indicated by black contour bands. Figure S2. Adjusted maps for (A) ASD prevalence (birth cohort n=11,034 and ASD n=532), (B) ASD-ID (birth cohort n=11,034 and ASD-ID n=318), and (C) ASD+ID (birth cohort n=11,034 and ASD+ID n=214). Maps are not significantly different than flat (global P=0.052, global P=0.294 and global P=0.196, respectively). Adjustment factors were year of birth; plurality; maternal age, race, and level of education; and report of tobacco use during pregnancy. [file 1476-069X-11-80-S1.docx]
